# Acute Incarceration of Solitary Rectal Ulcer Syndrome as an Anorectal Emergency in an Adolescent Patient: A Case Report With Long-Term Follow-Up and a Review of the Literature

**DOI:** 10.7759/cureus.117470

**Published:** 2026-10-05

**Authors:** Faisal Elagili, Norfaidhi Akram Mat Nor, Yusof Sainal, Muhammad Irfan Salmi, Azmi Md Nor

**Affiliations:** 1 Surgery, International Islamic University Malaysia Kulliyyah of Medicine, Kuantan, MYS

**Keywords:** adolescent, anorectal emergency, case report, incarceration, rectal prolapse, solitary rectal ulcer syndrome, transanal excision

## Abstract

Solitary rectal ulcer syndrome (SRUS) is an uncommon, chronic disorder of rectal mucosal prolapse that typically presents with rectal bleeding, mucus discharge, and straining, and can occasionally present as a polypoid mass rather than a discrete ulcer. Acute incarceration of SRUS through the anal canal, requiring emergency surgery, does not appear to have been previously reported. We describe an adolescent boy with a two-year history of biopsy-confirmed SRUS, managed conservatively with only partial symptomatic response, who presented with acute, irreducible incarceration of a large, actively bleeding polypoidal mass protruding through the anal canal. He underwent emergency examination under anaesthesia with complete transanal excision, achieving simultaneous diagnostic and therapeutic control, and histopathology confirmed SRUS without dysplasia or malignancy. He remained completely asymptomatic with no recurrence at long-term follow-up extending to nearly six years postoperatively. This case illustrates an apparently novel presentation of SRUS as an anorectal surgical emergency, supports complete transanal excision as effective definitive treatment, and is placed in the context of the wider published literature on polypoid and prolapse-related SRUS.

## Introduction

Solitary rectal ulcer syndrome (SRUS) was first described by Cruveilhier in 1829 [1] and later characterized in detail by Madigan and Morson in their landmark 1969 series [2]. It is an uncommon, chronic, benign disorder of the rectal mucosa, with an estimated annual incidence of approximately one per 100,000 population [3]. Although classically described in young adults, SRUS is reported across all ages, including children and adolescents [4,5].

Despite its name, SRUS is widely regarded as a diagnostic misnomer: lesions may be multiple rather than solitary, non-ulcerated, or frankly polypoid and can extend beyond the rectum into the sigmoid colon [1]. The unifying pathogenic mechanism is thought to be chronic rectal mucosal prolapse, whether overt or occult, combined with paradoxical puborectalis contraction during straining; this produces recurrent mechanical trauma, venous congestion, and mucosal ischaemia [6]. Additional contributory mechanisms proposed in the literature include rectal hypersensitivity, direct digital trauma, and chronic constipation [1,7].

Clinically, SRUS presents with rectal bleeding, mucus discharge, tenesmus, straining at defecation, and a sensation of incomplete evacuation; overt full-thickness rectal prolapse is comparatively uncommon [8]. Diagnosis rests on the combination of history, endoscopic appearance, and critically, histopathology, because the differential diagnosis includes inflammatory bowel disease, inflammatory cloacogenic polyp, and colorectal neoplasia, each of which can mimic SRUS both clinically and endoscopically [7,9,10]. A polypoid variant, in which SRUS presents as a discrete mass rather than as an ulcer, is recognized in up to 32% of biopsy-proven cases and is a recognized cause of diagnostic delay because of its resemblance to neoplastic, haemorrhoidal, or inflammatory polypoid disease [10,11].

We report a case in which chronic, biopsy-confirmed SRUS progressed, despite combined conservative, medical, and behavioural therapy, to acute incarceration of a giant polypoid mass through the anal canal, a presentation that, to the best of our knowledge, has not previously been described, with outcome followed to nearly six years after emergency surgical management, the longest such follow-up we identified for a surgically treated polypoid SRUS presentation in the published literature. We review the literature on polypoid and prolapse-related SRUS to place this presentation in context.

This article was previously presented as poster PP01 ("Acute Incarceration Prolapsed Solitary Rectal Ulcer: A Rare Case of Anorectal Emergency") at Virtual Coloproctology 2021, the Malaysian Society of Colorectal Surgeons annual scientific meeting, held on 4-7 March 2021.

## Case presentation

An 18-year-old adolescent boy presented to the emergency department with a three-hour history of an acutely incarcerated and actively bleeding mass protruding from the anal canal. He had no significant past medical history apart from a diagnosis of SRUS established two years earlier, when he had presented with a one-year history of constipation, straining during defecation, and per-rectal bleeding. He reported a long-standing habit of prolonged toilet sitting since childhood, consistent with a straining-related pathogenesis. There was no history of overt rectal prolapse. At that time, colonoscopy demonstrated a polypoidal growth located 6-10 cm from the anal verge, and biopsy confirmed SRUS. Since the diagnosis, he had been managed with laxatives, dietary modification, mesalamine enemas, and biofeedback therapy, with only partial symptomatic improvement. He had also required multiple hospital admissions and blood transfusions for bleeding-related anemia.

On the index presentation, the patient reported lethargy and dizziness consistent with acute blood loss. Admission investigations revealed a hypochromic, microcytic anaemia with an associated leukocytosis, neutrophilia, and lymphocytosis, and mild hyponatraemia; renal function was otherwise normal (Table 1). Vital signs were within normal limits, and he was haemodynamically stable; he did not require blood transfusion during this admission. Abdominal examination was unremarkable, with a soft, non-tender abdomen and no signs of peritonism.

**Table 1:** Laboratory investigations on admission

Test	Result	Reference range
Sodium	135	136-146 mmol/L
Potassium	3.6	3.5-5.1 mmol/L
Chloride	106	102-112 mmol/L
Urea	3.4	1.8-7.1 mmol/L
Creatinine	76	71-123 µmol/L
Haemoglobin	81	130-170 g/L
Haematocrit	0.26	0.40-0.50 L/L
Red blood cell count	3.9	4.50-5.50 × 10¹²/L
Mean corpuscular volume (MCV)	68	77-97 fL
Mean corpuscular haemoglobin (MCH)	20.8	27.0-32.0 pg
Mean corpuscular haemoglobin concentration (MCHC)	307	315-345 g/L
Red cell distribution width (RDW)	36	11.6-14%
White blood cell count	19.3	4.0-10.0 × 10⁹/L
Platelet count	292	150-400 × 10⁹/L
Neutrophils (absolute)	12.7	2.00-7.00 × 10⁹/L
Neutrophils (%)	65.5	-
Lymphocytes (absolute)	5.6	1.00-3.00 × 10⁹/L
Lymphocytes (%)	29.2	-
Monocytes (absolute)	0.9	0.20-1.00 × 10⁹/L
Monocytes (%)	4.6	-
Eosinophils (%)	0.3	-

Perianal inspection revealed a large, circumferential, cauliflower-like polypoidal mass, measuring approximately 15 × 10 cm, protruding through and obliterating the anal canal (Figure 1). The mass was irreducible and exquisitely tender; digital rectal examination could not be performed because of pain and luminal obstruction by the lesion. A trial of gentle external manual reduction was unsuccessful.

**Figure 1:**
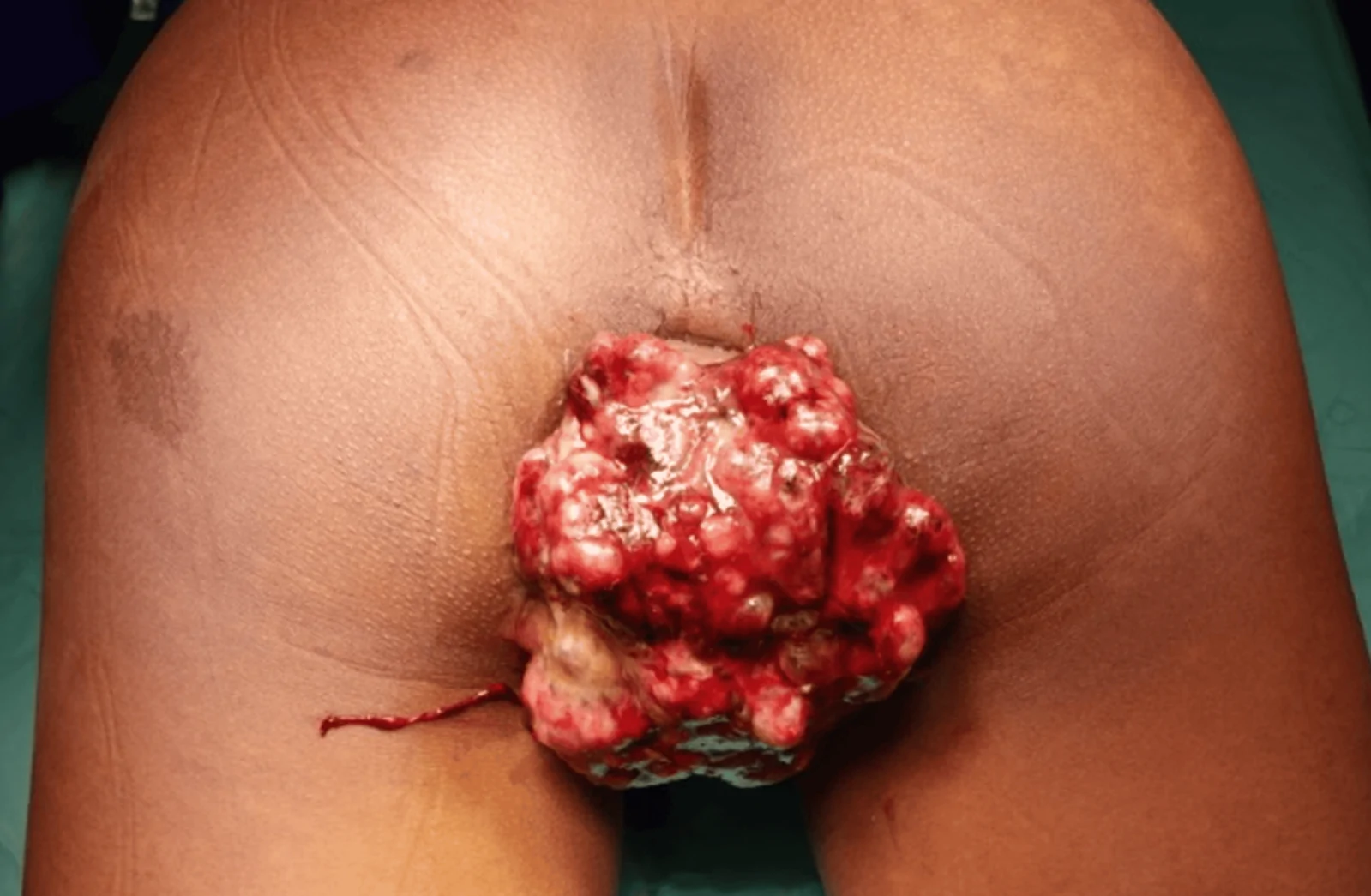
Clinical photograph at presentation showing a large, circumferential, cauliflower-like polypoidal mass (15 × 10 cm) incarcerated and protruding through the anal canal

Given the incarcerated, irreducible, and actively bleeding nature of the mass, the patient was taken to the operating theatre for emergency examination under anaesthesia (EUA). Under spinal anaesthesia, the mass was confirmed to represent circumferential prolapse of the known rectal SRUS lesion through the anal canal, obliterating the lumen; the tissue was friable and fragile. Because reduction could not be achieved and the tissue was incarcerated and actively bleeding, complete transanal excision of the polypoidal mass was performed (Figure 2), with hemostasis secured and the resulting defect closed primarily using 3-0 Vicryl; estimated blood loss was approximately 100 mL, and operative time was approximately one hour, achieving both diagnostic and immediate therapeutic control of the emergency.

**Figure 2:**
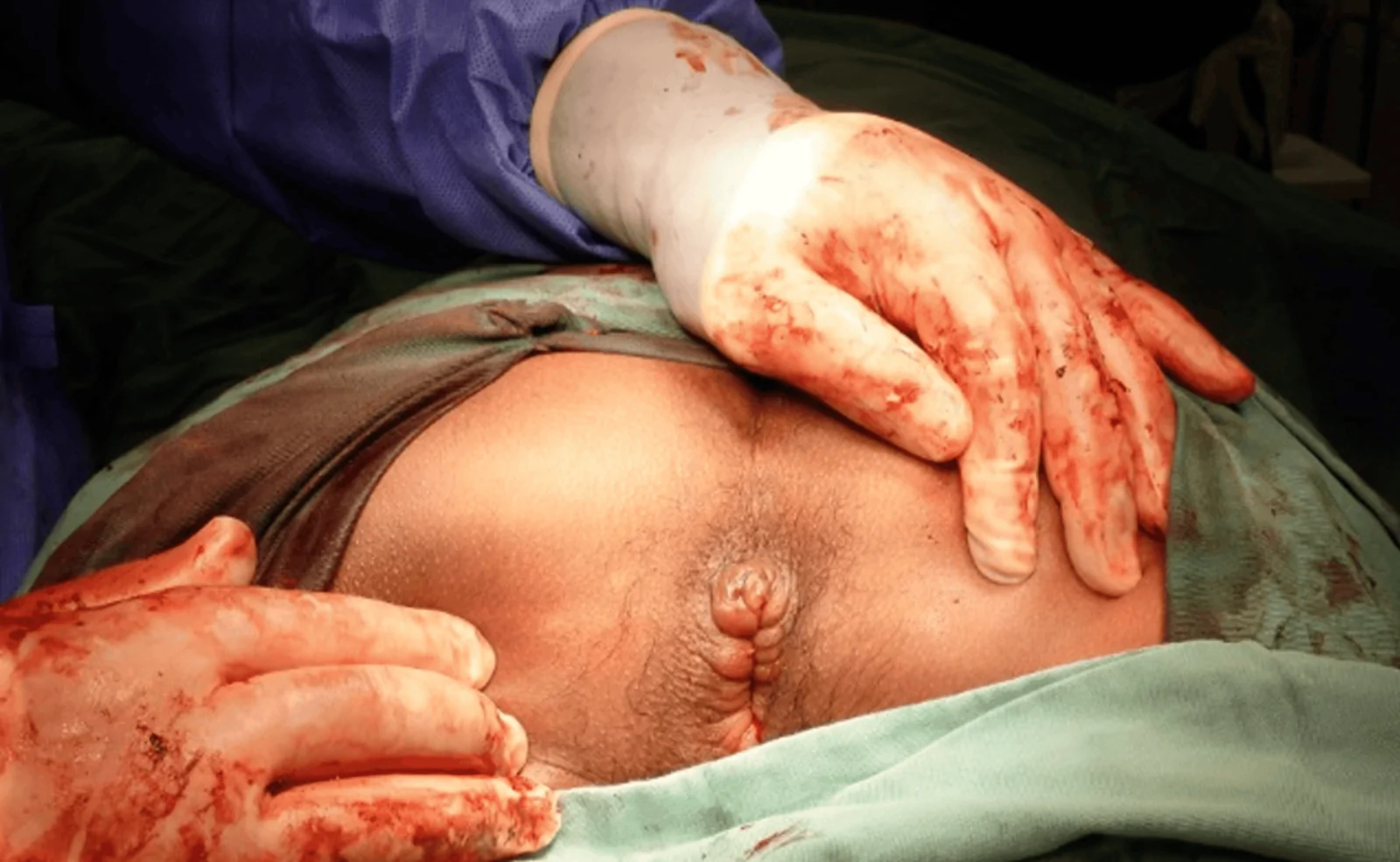
Intraoperative appearance following complete transanal excision of the polypoidal mass under spinal anaesthesia (EUA), with the anal canal restored EUA: examination under anaesthesia

Histopathological examination showed multiple polypoidal fragments with extensive surface ulceration and only focal residual intact mucosa lined by reactive epithelium. The lamina propria was oedematous and replaced by granulation tissue with a dense acute and chronic inflammatory infiltrate, haemorrhage, and inspissated mucin (Figure 3). There was variable fibromuscular hyperplasia with thickening, splaying, and vertical extension of the muscularis mucosae into the lamina propria (Figure 4), together with crypt architectural distortion and dilatation lined by benign mucin-secreting columnar epithelium showing reactive, non-dysplastic atypia. No malignancy, dysplasia, cryptitis, crypt abscesses, or granulomas were identified. These findings were consistent with SRUS.

**Figure 3:**
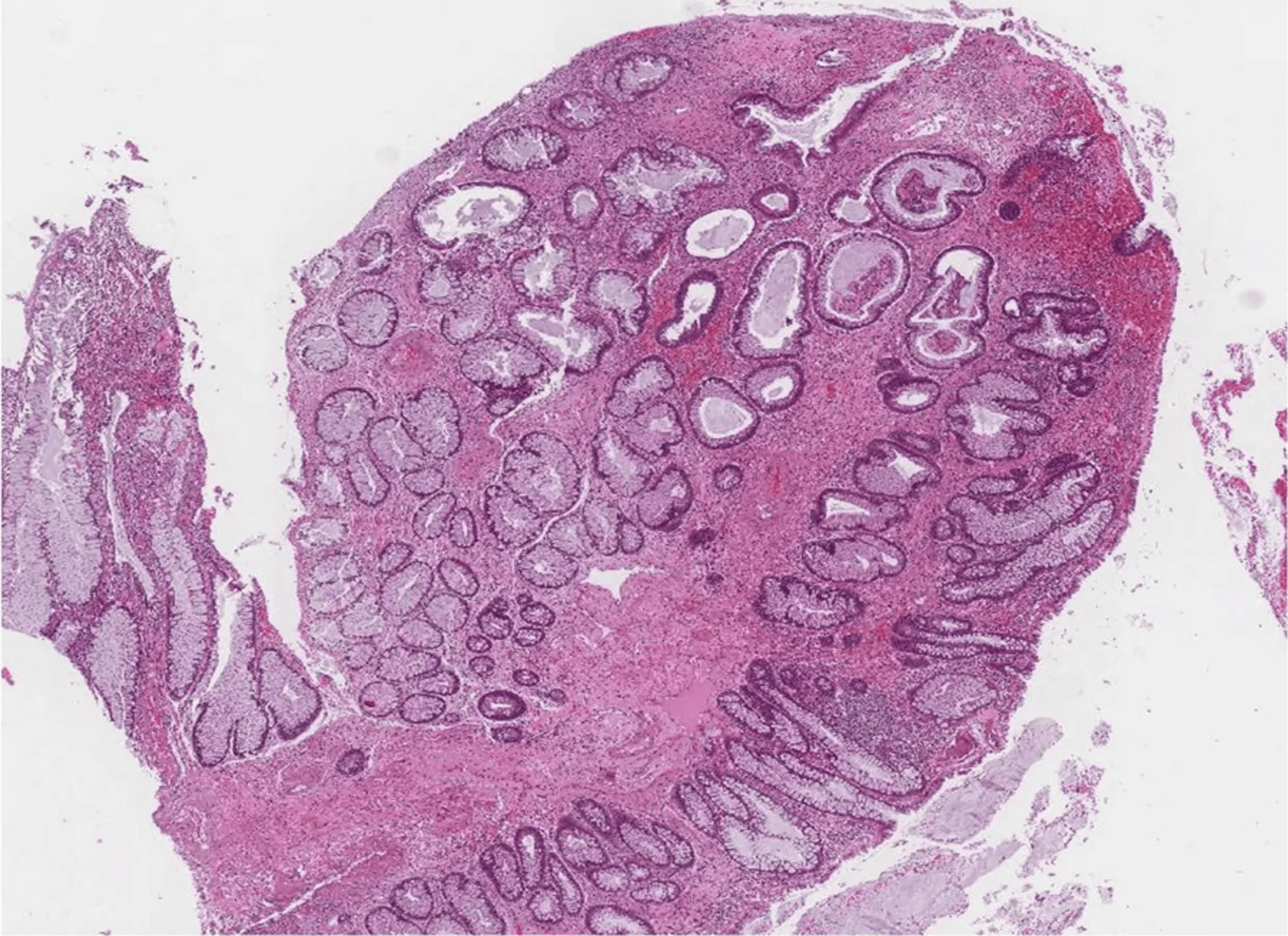
Photomicrograph (H&E stain, low power, 100x magnification) showing an ulcerated polypoidal mass with residual benign mucosal lining Crypt branching and dilatation are present, together with haemorrhage, granulation tissue, and fibromuscular hyperplasia within the lamina propria. H&E: haematoxylin and eosin

**Figure 4:**
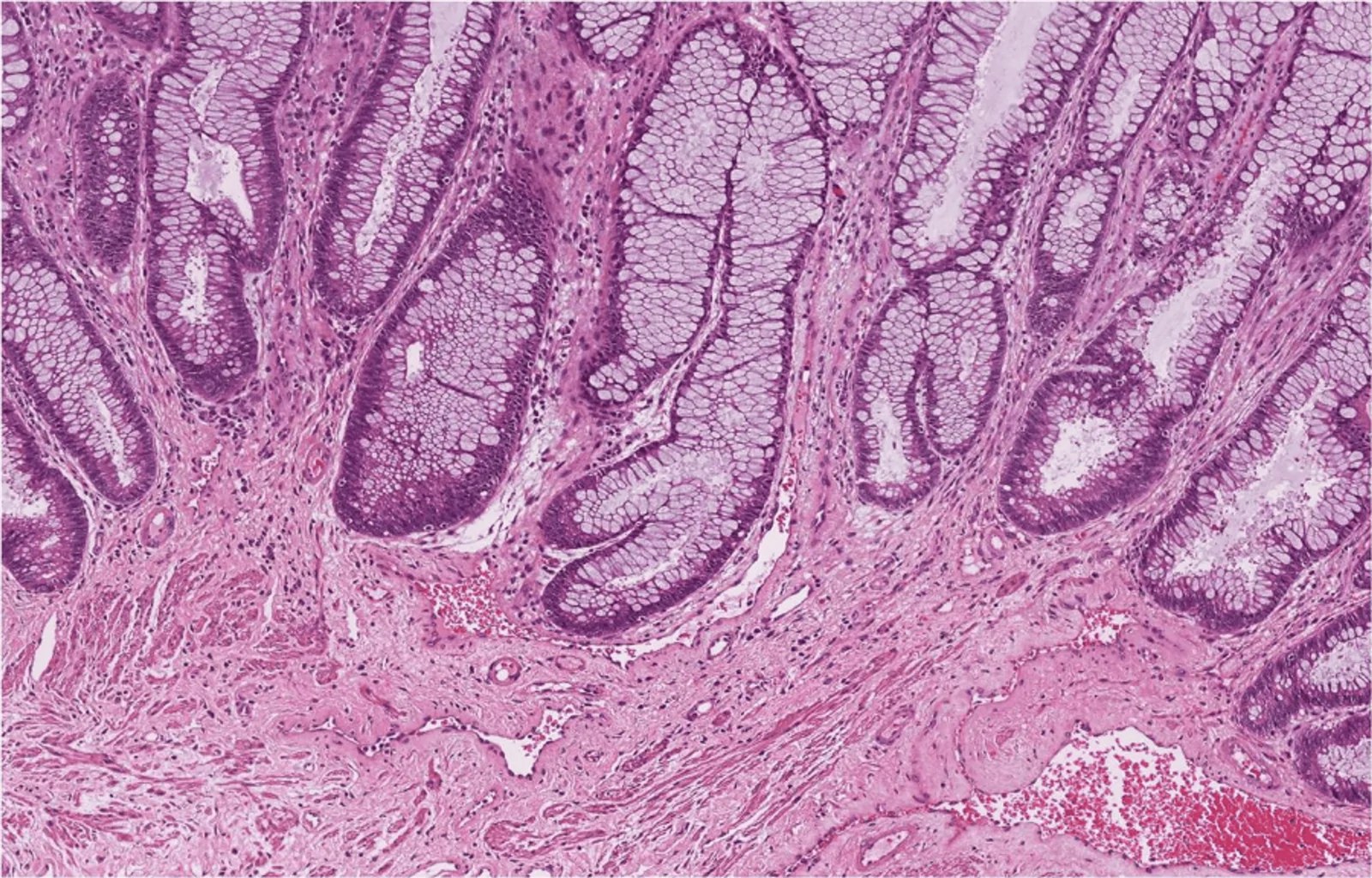
Photomicrograph (H&E stain, high power, 200x magnification) showing fibromuscular hyperplasia with thickening, splaying, and vertical extension of the muscularis mucosae into the lamina propriam Fibromuscular hyperplasia is a histological feature characteristic of solitary rectal ulcer syndrome (SRUS). H&E: haematoxylin and eosin

The patient's postoperative course was uneventful, and he was discharged on postoperative day three with advice on toileting behaviour and continuation of biofeedback therapy. He was subsequently reviewed in clinic and remained symptom-free at follow-up more than two years after surgery. At the most recent follow-up, approximately five years and nine months after the index operation, with the patient now 23 years of age, he remained completely asymptomatic, with no recurrence of bleeding, prolapse, or other bowel symptoms since surgery; anal sphincter function was intact, there was no anal stenosis on examination, and the patient denied incontinence.

## Discussion

We searched PubMed/MEDLINE (inception to 2026) using combinations of the terms "solitary rectal ulcer syndrome", "prolapse", "incarcerated", "strangulated", "irreducible", and "anorectal emergency", supplemented by hand-searching the reference lists of retrieved articles. Case reports and series describing SRUS presenting as a polypoid or prolapsing mass were reviewed in detail for direct comparison; reports of incarcerated or strangulated full-thickness rectal prolapse (procidentia) without confirmed SRUS histology represent a pathologically distinct entity and were excluded from direct comparison, since that condition and its literature are not equivalent to SRUS. This was a narrative, not a systematic, review; the search was limited predominantly to English-language, PubMed-indexed literature, which is stated explicitly as a limitation below.

SRUS is now understood as a disorder of chronic mucosal prolapse rather than a primary ulcerative disease. Womack et al. demonstrated radiologically that patients with SRUS exhibit a high incidence of rectal prolapse (94% in voiding studies) together with paradoxical overactivity of the external anal sphincter during evacuation, producing the elevated intrarectal pressures thought to drive mucosal ischaemia and ulceration [6]. Symptoms include per-rectal bleeding, mucus discharge, abdominal pain, tenesmus, constipation, and uncommonly overt rectal prolapse [3,7,12]. In a series of 28 patients, lesions were multiple in up to 34% and polypoidal or nodular, rather than classically ulcerative, in up to 32% [11]; in the largest reported endoscopic series (116 cases), a separate group similarly found a 25% polypoid/nodular proportion against 78% ulcerative lesions [10], and lesions may be located anywhere from 2-20 cm from the anal verge, most often on the anterior or anterolateral rectal wall [12].

Diagnosis is established on the combination of clinical features, endoscopic appearance, and histopathology [1,7]. Histologically, the defining triad is fibromuscular obliteration of the lamina propria, crypt architectural distortion, and disorientation of muscle fibres within a thickened, splayed muscularis mucosae; Al-Brahim et al. confirmed surface serration, crypt distortion, vascular ectasia, and fibromuscular obliteration in all 13 cases in their series [13]. The absence of cryptitis, crypt abscesses, and granulomas is supportive against inflammatory bowel disease, although focal acute inflammatory change can occur in SRUS and is not by itself discriminating; the overall histological pattern, taken together with the clinical course, was considered most consistent with SRUS rather than IBD. The absence of dysplasia or invasive features, as in the present case, is essential to exclude colorectal neoplasia. The polypoid variant of SRUS in particular must be distinguished from inflammatory cloacogenic polyp (a histologically closely related lesion arising at the anal transitional zone) [9], villous or tubulovillous adenoma, juvenile polyp, and, in the acute incarcerated setting specifically, from full-thickness rectal procidentia without SRUS features. Radiological adjuncts such as defecography and pelvic MRI have a role but are used less frequently now that endoscopy and biopsy are more readily accessible [8].

A structured comparison of the present case with the major published series and the most closely related individual case report is summarized in Table 2. Merali et al. described a 67-year-old man with a 12-year history of worsening rectal bleeding and mucus discharge, in whom examination under anaesthesia identified a pedunculated polypoidal lesion measuring 25 × 20 mm and protruding 50 mm from the anorectal junction; this was managed by excision biopsy in a planned, non-emergency setting, and histology confirmed SRUS [14]. That report, to our knowledge, the only prior case of a single, large, SRUS-confirmed polyp protruding from the anal canal, differs from the present case in three material respects: the presentation was chronic and progressive rather than acute; the lesion was neither incarcerated nor irreducible; and it was roughly a 15th of the surface area of the mass described here. It also differs in follow-up duration: that report documented eight weeks of uneventful follow-up, whereas the present patient has been followed to approximately five years and nine months postoperatively with sustained symptom resolution to our knowledge, the longest reported follow-up for a surgically managed polypoid SRUS presentation of this kind, and one that adds meaningful confidence regarding the durability of excision in this specific presentation.

Taken together, based on the search strategy described above, we are not aware of any previously published report describing acute, irreducible incarceration of biopsy-confirmed SRUS managed by emergency transanal excision. We propose that the present case represents, to the best of our knowledge, the first such report; however, given the inherent limitations of a narrative, predominantly English-language, PubMed-based search, we recommend that this claim be independently corroborated in Scopus and Embase before publication, since case reports in this specific niche are precisely the kind of material that a single-database search can miss.

Management of SRUS is generally stratified by symptom severity and the presence of prolapse. Conservative measures, patient education, a high-fibre diet, avoidance of straining, and regulation of toileting habits are first-line and are effective in mild-to-moderate disease without prolapse [1,3,8,11,15,16]. Biofeedback therapy has shown encouraging results: Jarrett et al. reported improved rectal mucosal blood flow and ulcer resolution in 31% of patients undergoing biofeedback [17]. Medical therapies, including topical sucralfate, mesalamine enema, and corticosteroids, have also been used; a systematic review and meta-analysis reported pooled ulcer resolution of 57% with medical treatment, though the authors emphasized substantial heterogeneity and the absence of randomized controlled trial evidence [18]. Surgery local excision, low anterior resection, rectopexy, Delorme's procedure, Altemeier's procedure, or faecal diversion is generally reserved for patients who fail medical and behavioural therapy or who have overt prolapse [8,16]. Reported surgical outcomes are conflicting: Marchal et al. described a high failure rate in a series with a mean follow-up of 4.5 years and recommended surgery be reserved for prolapse or truly intractable symptoms [15], whereas Bulut et al. reported symptomatic improvement in 78% of surgically treated patients [16]. It should be emphasized that all these outcome data derive from small, retrospective case series-level IV evidence by conventional hierarchies and that no randomized controlled trials exist for the surgical management of SRUS; management recommendations in this area are consequently of low-to-moderate certainty and should be individualized. In the present case, the pre-existing failure of biofeedback and medical therapy, combined with an emergency incarceration necessitating surgical control, justified proceeding directly to excision; the multimodal approach (biofeedback, medical therapy, and eventual surgical excision) is consistent with the stepwise strategy described in this literature.

This report has the inherent limitations of a single case, and its conclusions cannot be generalized. A particular strength, however, is the duration of follow-up: the patient has been followed to approximately five years and nine months postoperatively, with the most recent assessment confirming sustained symptom resolution substantially exceeding the follow-up reported for the only directly comparable published case [14] and adding confidence regarding the durability of excision in this specific presentation. At the most recent review, the patient remained symptom-free and digital rectal examination did not reveal a palpable mass, and repeat endoscopy was therefore not pursued as it was not clinically indicated. Our literature search, although structured, was narrative rather than systematic and was conducted predominantly through PubMed/MEDLINE with supplementary web-based searching; it is possible that additional relevant cases exist in Scopus, Embase, or regionally indexed journals, or in unpublished conference proceedings, that were not captured. The novelty claim made here should therefore be read as based on the evidence identified rather than as an absolute statement, and independent verification in additional databases is recommended before submission.

**Table 2:** Comparative analysis of the major published solitary rectal ulcer syndrome (SRUS) series and the most closely related individual case report discussed in this review, alongside the present case EUA: examination under anaesthesia

Study	N	Design	Key presentation/lesion features	Management	Outcome
Martin et al., 1981 [3]	51	Retrospective case series	Solitary ulcer in 35%; multiple ulcers 22%; polypoid lesions 25%; full-thickness prolapse to/beyond the anal verge in 59%	High-roughage diet/bulking agents; rectopexy in 6 with associated complete prolapse	Two-thirds improved with conservative measures; rectopexy results satisfactory in the followed cases
Chong and Jalihal, 2006 [11]	28 (14 M)	Retrospective case series	Bleeding per rectum in 86%; lesions polypoidal/nodular in 32.1%, ulcerative in 64.3%, multiple in 34%	Not detailed in report (supportive/medical care)	Persistent bleeding per rectum in 39%; 64% had multiple hospital admissions (mean 3.1), mainly for transfusion
Abid et al., 2012 [10]	116	Retrospective case series	Bleeding per rectum in 82%; lesions polypoidal in 25%, ulcerative in 78%, multiple in 28%	Not detailed in report (diagnostic/endoscopic series)	Not detailed in report; underlying neoplasia identified in 2 patients on work-up
Al-Brahim et al., 2009 [13]	13 (8 M/5 F)	Retrospective clinicopathological study	Rectal bleeding, constipation, and abdominal pain most common; 2 patients had associated rectal polyps	Not detailed in report (diagnostic/histological study)	Not detailed in report
Marchal et al., 2001 [15]	13	Retrospective surgical series	Associated internal rectal prolapse in 58%, total prolapse in 15%, mucosal prolapse in 15%	Delorme's procedure (62%), Orr-Loygue rectopexy (23%), simple resection or stoma (15%)	Improvement in 62.5% after modified Delorme's procedure; simple resection did not improve symptoms; mean follow-up 57 months
Bulut et al., 2011 [16]	58 (23 operated)	Retrospective case series	Subset with paradoxic rectal spasm trialled biofeedback/botulinum toxin before surgery	Surgical treatment in 23 patients who failed conservative measures	Postoperative improvement in 78.2% of surgically treated patients
Merali et al., 2017 [14]	1 (67/M)	Case report	Chronic rectal bleeding and mucus discharge, worsening over 12 years; pedunculated polypoidal lesion 25 × 20 mm, protruding 50 mm; not incarcerated, elective/urgent EUA	Excision biopsy at EUA	Uneventful; no recurrence at 8 weeks
Present case	1 (18/M, index presentation)	Case report	Acute, irreducible incarceration of known SRUS, 3-hour history, actively bleeding polypoidal mass, 150 × 100 mm	Emergency transanal excision	Uneventful; symptom-free at follow-up to ~5 yr 9 mo postoperatively (age 23)

## Conclusions

Acute incarceration of a polypoid SRUS lesion through the anal canal is, to the best of our knowledge, a previously unreported anorectal surgical emergency. This case demonstrates that chronic, treatment-refractory SRUS can progress to an acute, irreducible, actively bleeding incarceration requiring emergency surgical intervention, and that complete transanal excision, rather than the bowel resection typically required for incarcerated full-thickness rectal procidentia, can achieve definitive diagnostic and therapeutic control when the underlying pathology is a discrete polypoid lesion rather than full-thickness prolapse.

Follow-up to nearly six years postoperatively, with sustained complete symptom resolution, provides the longest reported outcome data for this presentation. In this patient, complete transanal excision achieved durable symptom control without recurrence; this single case observation cannot establish transanal excision as generally definitive treatment for polypoid SRUS incarceration, but it provides proof-of-concept evidence supporting its use as first-line surgical management in this specific clinical presentation, to be corroborated by future cases. SRUS should be included in the differential diagnosis of an incarcerated, prolapsing anal mass, particularly in a patient with a known prolapse-related diagnosis and a treatment-refractory course, once colorectal neoplasia and other prolapse-related mimics have been excluded.
